# Adherence of Baseline Physical Health Monitoring for Patients Receiving Antipsychotic Medications in a Psychiatry Ward, Lahore

**DOI:** 10.1192/bjo.2024.635

**Published:** 2024-08-01

**Authors:** Asma Suleman, Ali Madeeh Hashmi

**Affiliations:** Punjab Institute of Mental Health, Lahore, Pakistan

## Abstract

**Aims:**

- To identify current practices regarding baseline tests monitoring for antipsychotic medications.
- To identify potential causes for not adhering to the standard guidelines.
- To ensure all the baseline tests have been documented and reviewed properly.

**Methods:**

In a retrospective analysis, a cohort of 28 patient case notes was examined in June 2023 to assess the baseline physical health parameters within the Psychiatry department at Punjab Institute of Mental Health, Lahore, in accordance with the guidelines outlined in “The Maudsley Prescribing Guidelines in Psychiatry 13th edition.” The data was analyzed to fulfill the audit objectives, and significant trends were subsequently identified.

**Results:**

The baseline assessments encompassed a comprehensive blood count, urea and electrolyte analysis, liver function testing, blood glucose measurement, and blood pressure monitoring, all of which were conducted in 100% of the cases. Nevertheless, electrocardiography (ECG) was only carried out in 71% of the cases prior to the initiation of antipsychotic treatment. Regrettably, there was a lack of documentation regarding baseline weight/BMI monitoring, serum prolactin level assessment, and creatinine phosphokinase level measurement.

**Conclusion:**

The audit revealed several areas of concern that warrant immediate attention and improvement. These include:

- Protocols and Guidelines: The absence of defined protocols poses a significant challenge to maintaining consistent and standardized practices within the department.
- Awareness and Training: There is a noticeable lack of awareness among medical staff, including doctors and nurses, regarding the importance and proper procedures for baseline assessments.
- Sampling Errors: The occurrence of sampling errors during the data collection process has impacted the reliability and accuracy of the obtained results.
- Administrative Challenges: Administrative issues have been identified as a barrier to the seamless implementation of baseline assessment protocols.
- Resource Allocation: Insufficient funding for laboratory resources has hindered the comprehensive and timely conduct of essential tests.
- Test Availability: The limitations in the availability of certain required tests have impeded the thoroughness of baseline assessments for patients.

Addressing these areas of improvement is critical to enhancing the quality of care and ensuring the holistic well-being of our patients. It is imperative to implement robust protocols, enhance staff awareness and training, rectify administrative challenges, secure adequate funding for resources, and ensure the availability of essential tests. These measures will contribute to the delivery of comprehensive and effective healthcare services within the Psychiatry department at Punjab Institute of Mental Health, Lahore.

